# Conversion of biomass-derived sorbitol to glycols over carbon-materials supported Ru-based catalysts

**DOI:** 10.1038/srep16451

**Published:** 2015-11-18

**Authors:** Xingcui Guo, Jing Guan, Bin Li, Xicheng Wang, Xindong Mu, Huizhou Liu

**Affiliations:** 1 CAS Key Laboratory of Bio–based Materials, Qingdao Institute of Bioenergy and Bioprocess Technology, Chinese Academy of Sciences, Qingdao 266101, China

## Abstract

Ruthenium (Ru) supported on activated carbon (AC) and carbon nanotubes (CNTs) was carried out in the hydrogenolysis of sorbitol to ethylene glycol (EG) and 1,2-propanediol (1,2-PD) under the promotion of tungsten (WO_x_) species and different bases. Their catalytic activities and glycols selectivities strongly depended on the support properties and location of Ru on CNTs, owning to the altered metal-support interactions and electronic state of ruthenium. Ru located outside of the tubes showed excellent catalytic performance than those encapsulated inside the nanotubes. Additionally, the introduction of WO_x_ into Ru/CNTs significantly improved the hydrogenolysis activities, and a complete conversion of sorbitol with up to 60.2% 1,2-PD and EG yields was obtained on RuWO_x_/CNTs catalyst upon addition of Ca(OH)_2_. Stability study showed that this catalyst was highly stable against leaching and poisoning and could be recycled several times.

Concerns about the depletion of fossil fuels and the impacts of global warming issues result in increasing attention on the conversion of renewable biomass to fuels and chemicals^1^,^2^,^3^,^4^. For environmental and economic reasons, lignocellulose-derived sugar alcohols, such as sorbitol and xylitol, have emerged as the most potential building block chemicals.

Ethylene glycol (EG) and propylene glycol (1,2-PD) with annul consumption of over 20 million tons are industrially important chemicals used in the manufacture of polymers, resins, functional fluids, perfumes, cosmetics, etc^5^,^6^. Currently, they are industrially produced by multiple steps of cracking, epoxidation, and hydration from petroleum-derived ethylene and propylene, respectively^7^. To replace petroleum-based sources, the hydrogenolysis of sorbitol for the production of glycols is a hot topic^8^,^9^.

The selective hydrogenolysis of sorbitol into glycols such as EG, 1,2-PD and glycerol (GLY) was a challenge since complex parallel and consecutive C−C and C−O bond cleavage reactions occurred in aqueous medium, leading to a complex mixture of reactants, intermediates and products.

Many metals such as Ni, Cu, Ru, Pt and Pd-based catalysts have been used for the polyol hydrogenolysis reaction^10^,^11^,^12^, among them, Ru showed the highest catalytic activity. Sun and Liu^13^ found that activated carbon supported Ru catalyst exhibited higher activities and glycol selectivities than Ru on TiO_2_, ZrO_2_, Al_2_O_3_ and Mg_2_AlO_x_ supports for xylitol hydrogenolysis. Zhao *et al*.^14^ reported that carbon nanofibers (CNFs)-supported Ru catalyst displayed attracting catalytic performance in comparison with commercial activated carbon-supported Ru catalyst, thus leading to a significant increase in the selectivity to EG, 1,2-PD and GLY from 26.1% to 51.3% at 70–85% sorbitol conversions under 220 ^o^C and 8 MPa H_2_. However, the nano-scale CNFs will induce severe difficulty in catalyst separation and product purification for practical application.

Carbon nanotubes (CNTs) as a new kind of carbon materials offer interesting possibilities as supports for metal particles, due to the sp^2^ carbon-constructed surface, the excellent electron transport performance and the electronic interaction of active nanoparticles with the CNTs walls^15^. It was reported that Ru-based nanoparticles deposited on CNTs showed higher catalytic activity than those on other carriers, like alumina, silica, or even activated carbon (AC), for sorbitol hydrogenolysis^16^. Therefore, CNTs were considered a promising supporting material for sorbitol hydrogenolysis.

Recently, bimetallic catalysts have been reported for many heterogeneous catalysis reactions, because bimetallic catalysts showed great improvements in activity than their monometallic analogues due to the “synergistic” effects between the two metals^17^. The use of bimetallic catalysts for sorbitol hydrogenolysis reaction has been reported. Chaudhari *et al*.^18^ investigated the promoting effect of Re on the activity of Ru/C during sorbitol hydrogenolysis and found that addition of Re could significantly enhance the yields of 1,2-PD and EG.

In this work, we prepared a series of Ru catalysts using AC and CNTs as supports and compared their intrinsic activities and selectivities in sorbitol hydrogenolysis. Furthermore, the catalytic performance was tuned by controlling the location of metal particles on interior or exterior walls of CNTs. The effect of different basic promoters such as Ca(OH)_2_ and Ba(OH)_2_, and the modification of Ru/CNTs with WO_x_ were also thoroughly investigated during sorbitol hydrogenolysis.

## Results

### Catalyst characterization

**Figure 1:**
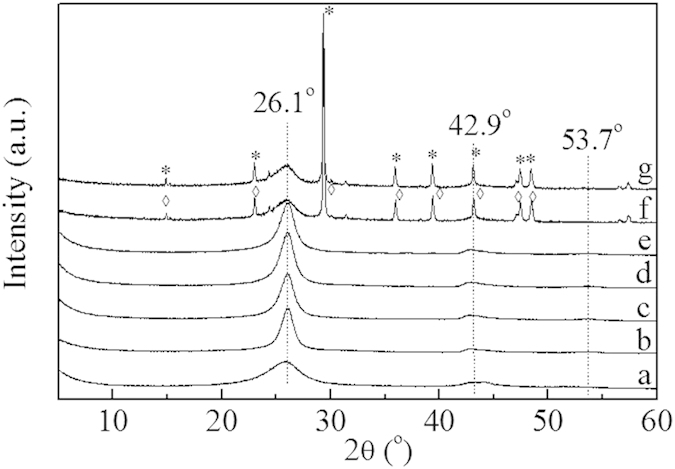
XRD patterns of (**a**–**e**) fresh and (**f**,**g**) spent catalysts: (**a**) Ru/AC; (**b**) Ru/CNTs-in; (**c**) Ru/CNTs-out; (**d**) Ru/CNTs; (**e**) RuWO_x_/CNTs; (**f**) Ru/CNTs-used and (**g**) RuWO_x_/CNTs-used.

The XRD patterns of Ru catalysts with different supports after the reduction at 350 ^o^C are displayed in Fig. 1. The diffraction peaks at around 26.0, 42.9 and 53.7^o^ were assigned to (002), (100) and (004) diffraction lines of raw CNTs, respectively^19^. No characteristic signals related to Ru species or WO_x_ particles were observed for all the samples, indicating that Ru or WO_x_ particle sizes on the supports were below the XRD detection limit, when the loadings of Ru and WO_x_ were lower than 10 wt%.

**Figure 2:**
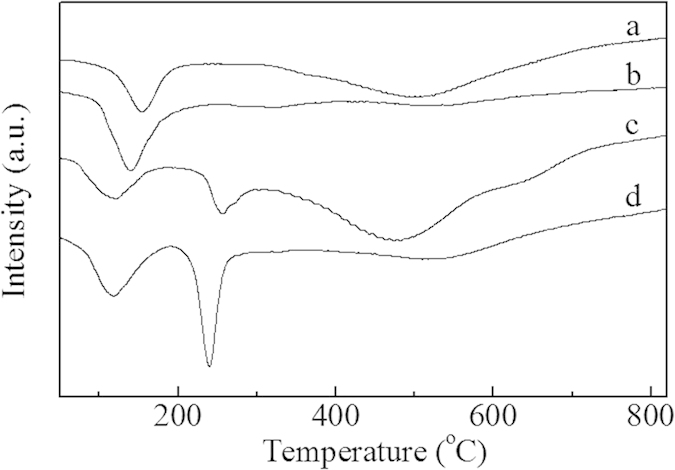
H_2_-TPR profiles for the Ru catalysts: (**a**) Ru/CNTs-in; (**b**) Ru/CNTs-out; (**c**) Ru0.25WO_x_/CNTs; (**d**) Ru0.50WO_x_/CNTs.

As displayed in Fig. 2, H_2_-TPR profiles for the samples Ru/CNTs-in and Ru/CNTs-out showed two reductive peaks centered at about 100–300 ^o^C and 500 ^o^C, respectively. The low temperature peak was likely due to the reduction of Ru^3+^ species to metallic Ru. The high temperature peak might be assigned to the reduction of carbon species on the surfaces of CNTs^20^. It was noted that Ru/CNTs-in sample had lower Ru^3+^ species reduction peak than Ru/CNTs-out, implying that the confinement of Ru oxide inside the CNTs pore resulted in easier reduction due to the shifting of π electron density from the inner to the outer surface caused by the deviation of the graphene layers from planarity^21^. As displayed, a new H_2_ consumption peak appeared for the WO_x_-containing catalysts at ca. 250 ^o^C. Clearly, this peak only observed for RuWO_x_/CNTs catalysts was assigned to the reduction of WO_x_ species^22^. Moreover, the intensity of the reduction peak increased with the increasing WO_x_ loading (Fig. 2d), suggesting a significant amount of reducible species was present on the modified catalyst.

**Figure 3:**
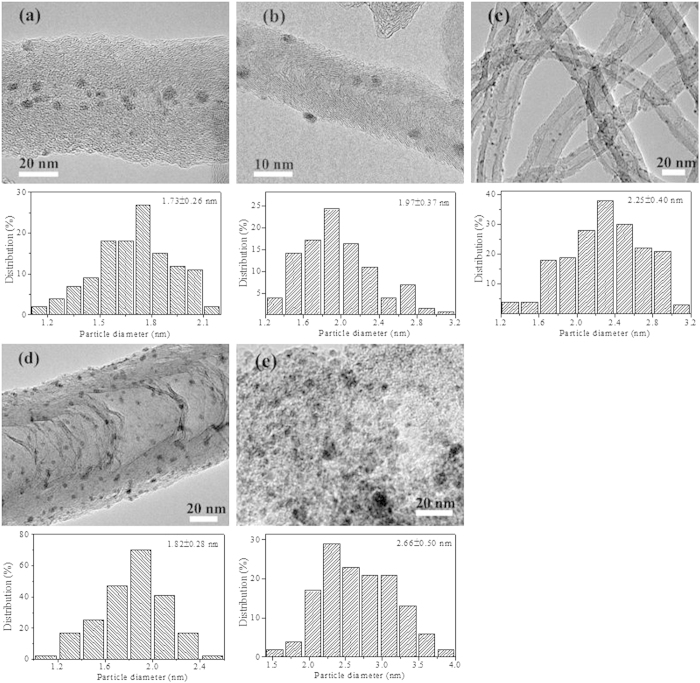
HRTEM micrographs and histograms of Ru particle size distribution for different Ru catalysts. (**a**) Ru/CNTs-in; (**b**) Ru/CNTs-out; (**c)** Ru/CNTs; (**d**) RuWO_x_/CNTs; (**e**) Ru/AC.

The average Ru particle size and distribution of the as-prepared catalysts were also characterized by using HRTEM. It can be seen from Fig. 3 that, aggregation of Ru nanoparticles was minimal, and Ru nanoparticles were highly dispersed with the average size of 2.0–3.0 nm for all the samples. For Ru/CNTs-in (Fig. 3a), the majority of metal particles were well distributed inside the nanotubes with an average size of 1.73 ± 0.26 nm. While for Ru/CNTs-out (Fig. 3b), Ru particles were found predominantly deposited on the exterior of the tubes with an average size of 1.97 ± 0.37 nm. From the histogram results, it could be observed that Ru particles deposited on the inside surface of CNTs were slightly smaller than that on the outside of the CNTs. This could be attributed to the strong metal-support interaction between the metal site with the inner surface of the CNTs which might prevent metal species from aggregating^23^.The as-prepared Ru/CNTs catalyst had a narrow Ru size distribution in the range of 1.85–2.65 nm as determined by TEM. The HRTEM images of RuWO_x_/CNTs (Fig. 3d) reveal homogeneous distribution of the metal nanocrystals with the average particle sizes of 1.82 nm. The Ru nanoparticle sizes of Ru/AC sample had a relatively wider size distribution, as shown in Fig. 3e and the mean nanoparticle sizes (diameter) were 2.66 ± 0.50 nm.

**Figure 4:**
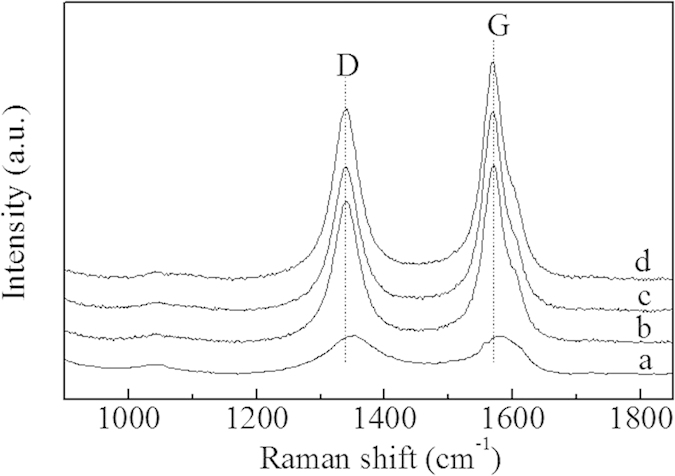
Raman spectra of (**a**) Ru/AC; (**b**) Ru/CNTs; (**c**) Ru/CNTs-in and (**d**) Ru/CNTs-out with 532 nm excitation wavelength.

Raman spectra of Ru/AC and Ru/CNTs, obtained with the 532 nm laser excitation line, are displayed in Fig. 4. The analysis of the peak positions and intensities give information about the changes of the structural characteristics of the samples. Each exhibited two characteristic bands, namely, the G-band at 1550–1600 cm^−1^ originating from the high degree of symmetry and order of carbon materials in graphene sheets, the D-band at 1250–1450 cm^−1^ attributed to the disordered graphite structure^24^. The intensity ratio of D and G bands (*I*_D_/*I*_G_) was an indicator of the degree of disorder with the samples^25^. Figure 4 presents that the *I*_D_/*I*_G_ ratios for Ru/AC, Ru/CNTs, Ru/CNTs-in and Ru/CNTs-out were 0.92, 0.72, 0.68 and 0.63, respectively. Ru/AC sample exhibited a pair of relatively broad peaks at about 1350 and 1579 cm^−1^, corresponding to the D- and G-bands. A high intensity ratio of D-bands to G-bands for Ru/AC implied a typical of amorphous carbons, indicating that its graphitic domains were much smaller in comparison with CNTs^26^. These results coincided with the observations from HRTEM.

**Figure 5:**
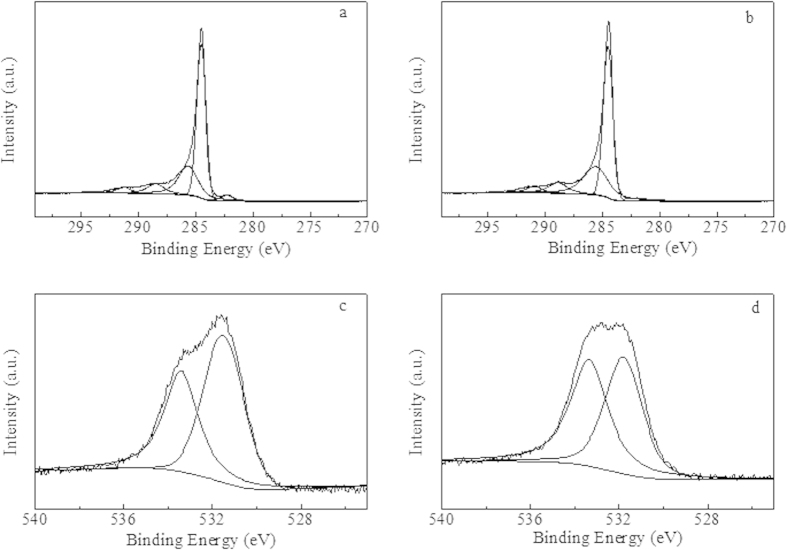
Ru 3d (**a**,**b**) and O1s (**c**,**d**) of XPS profiles for Ru/CNTs-out (**a**,**c**) and Ru/CNTs-in (**b**,**d**) catalysts.

Figure 5 shows the XPS survey scans of Ru/CNTs-in and Ru/CNTs-out after reduction in hydrogen at 350 ^o^C. One distinct sharp Ru (3d) peak around 281.2 eV was clearly observed on Ru/CNTs-out (Fig. 5a). This was typical value for zero-valence Ru, indicating that the deposited Ru was metallic. Compared with the sample of Ru/CNTs-out discussed above, the peak at 281.2 ± 0.2 eV corresponding to Ru (3d) was disappeared for Ru/CNTs-in sample (Fig. 5b) which might be shielded by the 3–5 nm thick CNTs walls, indicating Ru/CNTs-out could offer more Ru particles on the surface than the Ru/CNTs-in sample. Additionally, no obvious signals characteristic of Cl species were observed for both of the samples, showing that Cl species were removed completely from RuCl_3_ after the HNO_3_ treatment and followed reduction in hydrogen at 350 ^o^C.

BET surface, metal loading, dispersion and average metal particle sizes obtained from N_2_ physisorption and TEM measurements of all the catalysts are shown in Table S1. As could be seen, Ru/AC had higher BET surface areas in comparison with CNTs-supported Ru catalysts. It should be noted that the Ru particle size for Ru/AC sample was higher than those supported on CNTs, as measured by HRTEM. On the other hand, the value of average particle size over Ru/CNTs-in was similar to that of Ru/CNTs-out, in spite of the fact that the former exhibited higher dispersion values than the latter as measured from H_2_ chemisorption.

### Catalytic activity measurement

#### Hydrogenolysis of sorbitol over Ru/AC and Ru/CNTs

Table 2 shows the catalytic results obtained over a series of supported Ru catalysts in the hydrogenolysis of sorbitol. When Ru/AC was used as a catalyst in this system, 88.6% of sorbitol was consumed in 2 h with 41.5% selectivity for 1,2-PD and EG (Table 1, entry 1). For comparison, the activity of Ru/CNTs was tested under the same conditions and it was found that Ru/CNTs showed remarkably high catalytic activity, with 99.2% conversion of sorbitol and 55.2% yield for 1,2-PD and EG (entry 2). In general, this might be dependent on the dispersion of Ru catalysts and the different properties of supports.

However, HRTEM images of the different Ru catalysts showed that the Ru particles were distributed uniformly on the supports for all the five samples. The mean Ru particle sizes in Ru/CNTs and Ru/AC (Fig. 3c,e) samples were 2.25 nm and 2.66 nm, respectively, indicating that the Ru species prepared by impregnation method were almost the same. From these results, we could conclude that the intrinsic properties of the supports might play an important role in determining the activity of Ru catalysts for the hydrogenolysis of sorbitol in water.

From the element analysis as shown in Table S2 (Supplementary), it could be seen that the catalytic performance was strongly affected by the impurities content in Ru/AC catalyst. There were larger amounts of impurities (such as Cl^−^, SO_4_^2−^, PO_4_^3−^) on AC than CNTs. These electron-withdrawing elements would cause negative effects on the electronic structure of Ru catalysts for sorbitol hydrogenolysis, which leaded to the decrease in sorbitol hydrogenolysis activity greatly. On the other hand, the catalytic activities could be influenced by the electron conductivities for carbon materials supported Ru catalysts. As displayed in the HRTEM images (Fig. 3), CNTs showed a higher degree of graphitization than AC, indicating that CNTs had a higher electron conductivity than AC^27^,^28^, which made electron transfer from CNTs to ruthenium easier. This might be another reason for the increased activity of Ru/CNTs than Ru/AC.

#### Ru/CNTs-in and Ru/CNTs-out catalysts

The catalytic performance of Ru/CNTs-in and Ru/CNTs-out catalysts were tested in a stainless-steel autoclave reactor at 205 ^o^C and 5.0 MPa, and the results were summarized in Table 1 (entries 3 and 4). With the same Ru loading of 4%, the Ru/CNTs-in catalyst yielded 98.3% sorbitol conversion after 2 h with 28.5 and 18.4% yields to 1,2-PD and EG, respectively. The Ru/CNTs-out catalyst achieved almost complete sorbitol conversion with 58.5% combined yields towards 1,2-PD and EG. The XRD result and TEM measurement showed that the Ru particles located inside of the CNTs were distributed homogeneously which was similar to the dispersion of Ru/CNTs-out catalyst (Supplementary Table S1). So the activity difference between Ru/CNTs-in and Ru/CNTs-out could be ascribed to the different positions of Ru species. Guo *et al*.^29^ reported that the outside Ru exhibited a higher electron density than the inside Ru as revealed in the HRTEM characterization and first-principles calculations. Gallegos-Suarez *et al*.^30^ observed that electron rich Ru species (Ru ^δ−^) could favor the cleavage of the C=O bond and promote the hydrogenolysis of polyol into PD and EG. The dehydrogenation of polyol to the corresponding aldehyde or ketone intermediate on the metal catalysts was an electrophilic process. The outside Ru catalyst exhibited a high electron density should facilitate adsorption and activation of H_2_ or sorbitol, thus might be the reason for the enhanced sorbitol hydrogenolysis activities over Ru/CNTs-out in comparison with Ru/CNTs-in, as observed in Table 1.

#### Effects of promoters (WO_x_) on selective hydrogenolysis of sorbitol

The catalytic performance RuWO_x_/CNTs catalysts (with molar ratio of W/Ru of 0.25 and 0.50) in the sorbitol hydrogenolysis is given in Table 1. It could be found that addition of WO_x_ to Ru/CNTs enhanced the hydrogenolysis activity of sorbitol and glycols yields significantly (Table 1, entry 5). Ru0.25WO_x_/CNTs catalyst displayed a strong synergistic effect resulting in approximately 99.6% of sorbitol conversion with a 60.2% of total yield of products (Y_1,2-PD_ = 34.6%, Y_EG_ = 25.6%). However, further increase in molar ratio of WO_x_/Ru to 0.50, the conversion of sorbitol declined to 79% and over which only 46.4% glycols yields (Y_1,2-PD_ = 28.1%, Y_EG_ = 18.3%) was obtained. This was due to the fact that Ru metal surface was partially covered by WO_x_ cluster when doping much WO_x_ to Ru/CNTs catalysts^31^. H_2_-TPR profiles of Ru/CNTs and Ru–WO_x_/CNTs catalysts are also displayed in Fig. 2. It was found that the incorporation of WO_x_ component could remarkably increase the Ru species dispersion as indicated by the average Ru particle sizes on Ru/CNTs and RuWO_x_/CNTs. The HRTEM results (Supplementary Fig. S3) suggested that the presence of WO_x_ could also prevent Ru metal particles aggregation during the reaction, thus enhancing the selectivity of sorbitol towards the glycols.

#### Alkali promoter effect on Ru/CNTs catalysts

As reported before^32^, the activity of sorbitol hydrogenolysis over Ru/CNTs catalyst depended strongly on the basicity of catalyst. Sorbitol hydrogenolysis on a Ru catalyst in a basic medium follows a retro-aldolreaction mechanism which yields C2-C3 products such as EG, 1,2-PD, and GLY. The effect of base type on sorbitol hydrogenolysis was investigated by using various solid bases (i.e. Mg(OH)_2_, Ca(OH)_2_, Sr(OH)_2_ and Ba(OH)_2_) with equivalent theoretical OH^−^ and the results are listed in Table 2. It can be seen that all the bases could enhance the sorbitol hydrogenolysis activity of Ru/CNTs catalyst.

Alkaline earth metal hydroxides were weaker bases compared to their corresponding oxides, and the order of the strength of the basic sites was Ba(OH)_2_ > Sr(OH)_2_ > Ca(OH)_2_ > Mg(OH)_2_. The activities of Ru/CNTs catalyst for sorbitol hydrogenolysis with these promoters decreased in the order of Ca(OH)_2_ > Sr(OH)_2_ > Ba(OH)_2_ > Mg(OH)_2_, which was reversed to the strength of the basicity except Mg(OH)_2_. Among the four alkaline earth metal hydroxides examined above, Ca(OH)_2_ was clearly the most preferable one, leading to sorbitol conversion at above 99.2% and yield to glycols at 55.2% after 2 h (Table 2). In the case of Mg(OH)_2_, the conversion of sorbitol was 84.4% after 2 h reaction with a 1,2-PD yield of 11.5% and EG of 9.0%, and the major byproducts in this reaction were found to be glycerol (27.1%), mannitol (3.6%) and erythritol (6.9%) species. Some other gas products such as CO_2_, CH_4_, C_3_H_8_, n-butane, iso-butane, n-hexane and so on were also confirmed by GC-MS. This was possibly because of the low solubility of Mg(OH)_2_ in this reaction system.

According to the analyses above, the improved catalytic activity of the Ru-based catalysts in the presence of alkali promoters might be due to the fact that they could serve either as a Cl scavenger in the containing Ru catalysts, or as a modifier of the surface electronic states of Ru. The increased electron density of Ru catalyst facilitated dehydrogenation of polyols to the corresponding aldehyde or ketone intermediate. On the other hand, as a base additive, it provided a moderate basic medium for accelerating a retro-aldol reaction in the cleavage of C–C bond. However, an overly high OH^−^ strength also enhanced reversible and scrambling aldol reactions, resulting in a decline in selectivities to desired EG and 1,2-PD and a rise in yield of other undesirable hydrocracked products^33^.

##### Selectivity and Stability

Figure S1 (Supplementary) displays the change of selectivities for the three major products (1,2-PD, EG and GLY), as a function of the sorbitol conversions on Ru/CNTs and RuWO_x_/CNTs. It was shown that the selectivities for the three products strongly depended on sorbitol conversion and catalyst properties. The RuWO_x_/CNTs catalyst gave higher selectivities of 1,2-PD and EG compared with Ru/CNTs catalyst under the same conversion. On the RuWO_x_/CNTs catalyst, the selectivities to 1,2-PD increased from 16.4 to 34.7% with increasing the sorbitol conversion from 18.5 to 99.6%, meanwhile, the EG selectivities increased from 12.9 to 25.7%. Such trend led to the increase in the combined selectivity to 60.4% for the target glycols at nearly 100% sorbitol conversion, presenting the potential advantage of the RuWO_x_/CNTs catalyst for the selective hydrogenolysis of sorbitol. For Ru/CNTs catalyst, the selectivities to 1,2-PD increased from 14.4 to 31.6% with increasing the sorbitol conversion from 17.9 to 99.2%, and the selectivities to EG increased from 10.9 to 21.9%. As a consequence, the total selectivity for these two glycols reached 53.5% at almost completely sorbitol conversion. In contrast, the selectivities to GLY slightly declined for both RuWO_x_/CNTs and Ru/CNTs catalysts with increasing sorbitol conversion, which might be caused by the further conversion of GLY to glycols, as previously reported for the hydrogenolysis of glycerol over Ru-based catalysts^36^,^37^.

Figure S2 (Supplementary) shows the catalytic results for Ru/CNTs and RuWO_x_/CNTs in the hydrogenolysis of sorbitol with H_2_ under the identical reaction conditions in the presence of Ca(OH)_2_ through 5 repeated runs with regeneration. For Ru/CNTs catalyst, as illustrated in Fig. S3a (Supplementary), the Ru species of spent Ru/CNTs after five cycles increased from 2.25 to 3.25 nm. Thus, the activity decline was probably due to the partial agglomeration of Ru species caused by high pressure and the liquid phase nature of the reaction. Clearly, RuWO_x_/CNTs showed higher sorbitol conversion and glycols yields for all the runs than Ru/CNTs. As shown in Fig. S3b (Supplementary), the sizes of the Ru particles (being around 2.46 nm) changed slightly after the sorbitol hydrogenolysis reaction, indicating its exhibited better stability than Ru/CNTs catalyst upon reuse during the process of reaction. This was supported by the fact that no leaching of Ru or WO_x_ was detected by ICP and no clear lines attributed to Ru or WO_x_ species was revealed by XRD measurements for both the spent catalysts. The relatively higher stability of RuWO_x_/CNTs could be attributed to the synergy effect between Ru and WO_x_. Doping WO_x_ to Ru/CNTs catalysts could increase Ru dispersion and suppress the aggregation of Ru metal particles remarkably, but characteristic diffraction peaks of CaCO_3_ at 2θ = 23.04^o^, 29.40^o^, 36.00^o^, 36.40^o^, 43.16^o^, 47.48^o^ and 48.50^o^ were found for Ru/CNTs and RuWO_x_/CNTs after the fifth run, as revealed by XRD analysis (Fig. 1f,g). This demonstrated that part of the Ca(OH)_2_ was transformed into CaCO_3_ in the hydrogenolysis reaction.

## Discussion

The catalytic hydrogenolysis of sorbitol to 1,2-PD and EG under mild reaction conditions was investigated. It was found that support properties as well as position of Ru on CNTs had significant effects on the catalytic performance. The Ru/CNTs showed higher catalytic activity for sorbitol hydrogenolysis than Ru/AC which might be due to the higher graphitization degree and electron conductivities of CNTs. Ru nanoparticles dispersed on the outside surfaces of CNTs exhibited a higher activity than the CNTs-confined Ru. Addition of WO_x_ to Ru/CNTs was efficient in improving the catalytic performances and inhibiting the aggregation of Ru metal particles due to the synergistic effect between Ru with WO_x_. The suitable molar ratio of WO_x_/Ru was 0.25. Almost 100% conversion of sorbitol and above 60% glycols yields was obtained over Ru0.25WO_x_/CNTs catalyst at 205 ^o^C under 5.0 MPa. Importantly, this catalyst was structurally stable and showed excellent reusability.

## Methods

### Materials

Sorbitol, 1,2-PG, EG, Mg(OH)_2_, Ca(OH)_2_, Sr(OH)_2_ and Ba(OH)_2_ were purchased from Aladdin Industrial Inc. Commercial activated-carbon (AC), and multi-walled carbon nanotubes (CNTs, length: 0.5–2 μm,ID:5–10 nm, OD: 10–20 nm) were obtained from Chengdu Organic Chemicals Co., LTD and used as supports, Ruthenium chloride hydrate (RuCl_3_·xH_2_O) and ammonium tungstate ((NH_4_)_10_H_2_(W_2_O_7_)_6_) were purchased from Sinopharm Chemical Reagent Co., Ltd. (Shanghai, China) and used as precursors of ruthenium and tungsten, respectively.

### Catalyst preparation

Ru/AC and Ru/CNTs catalysts were prepared by the incipient wetness impregnation method using an aqueous RuCl_3_ solution as the Ru precursor, AC and CNTs as supports, respectively. After impregnation and subsequent evaporation of water under stirring, the samples were dried overnight at 110 °C, and then followed by reduction in a flow of H_2_/Ar at 350 °C for 3 h.

The RuWO_x_/CNTs was prepared by impregnating the dried Ru/CNTs in aqueous solutions of (NH_4_)_10_H_2_(W_2_O_7_)_6_. The catalyst was reduced in H_2_ at 350 °C for 3 h after drying at 110 °C for 12 h. The molar ratio of the WO_x_ to metal ruthenium (WO_x_/Ru) was 0.25 and 0.50, respectively.

Ru/CNTs-in and Ru/CNTs-out catalysts were prepared as described previously with some modifications^34^. In order to incorporate the Ru species into the channel of CNTs, the as-received raw CNTs were sonicated in concentrated HNO_3_ (68 wt%) at 40 ^o^C for 2 h. The mixture was then refluxed at 140 ^o^C for 12 h, washed with deionized water, and dried at 60 ^o^C for 10 h. Oxidized MWCNTs were dispersed in the acetone solution of RuCl_3_ utilizing the capillary forces aided by ultrasonication. After that the mixture was dried at 90 ^o^C for 10 h under a vacuum, followed by reduction in H_2_ at 350 ^o^C for 4 h. The final powder was labeled as Ru/CNTs-in catalyst.

For Ru/CNTs-out catalyst, the pretreated CNTs were impregnated in xylene with ultrasonication for 4 h. An aqueous solution of RuCl_3_ was then added, followed by addition of a solution of NH_4_HCO_3_ dissolved NH_3_·H_2_O. After stirring at 80 ^o^C for 0.5 h, the sample was subjected to the same drying and reduction treatment as previously. All the catalysts were prepared using RuCl_3_·xH_2_O as active component precursor with a nominal Ru loading of 4wt%.

### Catalyst characterization

Powder X-ray diffraction (XRD) patterns of catalyst samples were recorded on a D8 Advance X-ray diffractometer (Bruker, Germany) operated with Cu K irradiation and 2θ ranged from 10^o^ to 80^o^. Temperature programmed reduction by H_2_ (H_2_-TPR) measurements were conducted in an Auto Chem. II 2920 equipment (Mircromeritics, USA). The High Resolution Transmission Electron Microscopy (HRTEM) images were taken for determination of particle size on a JEOL-2100F microscope operated at 200 KV. For Raman spectroscopy tests, the 532-nm line from a Kimmon IK3201 R-F He-Cd laser was used for excitation with an intensity of ~20 Mw measured at the source. X-ray photoelectron spectroscopy (XPS) was performed using a PHI-560 ESCA (Perkin Elmer) spectrometer equipped with an Mg Kα target. A Thermo IRIS Intrepid II XSP atomic emission spectrometer was applied to determine the chemical composition of catalysts and metal leaching after reaction. The Brunauer–Emmett–Teller (BET) surface areas of the catalysts were carried out by the N_2_ physisorption technique with an apparatus (Micromeritics Tristar 3020). ICP analyses were carried out on a Thermo IRIS Intrepid II XSP atomic emission spectrometer to determine the chemical composition of catalysts and to examine metal leaching during reactions.

### Catalytic tests and analytical method

A general procedure to conduct the sorbitol conversion was the same as reported before^35^. The catalytic performance was examined in a 100 mL stainless steel autoclave equipped with a mechanical stirrer. For a typical test, known amounts of sorbitol aqueous solution and alkali were charged to the reactor along with an appropriate amount of catalysts. The reactor was purged three times with H_2_ at 5 MPa and heated up to the desired temperature for a given period at 500 rpm stirring speed.

The obtained products were quantified by Agilent 7890A gas chromatography coupled with Dionex ICS-3000ion chromatography. The total carbon balance (TC) was also measured on a liqui TOC II analyzer (Elementar Analysensysteme).

## Additional Information

**How to cite this article**: Guo, X. *et al*. Conversion of biomass-derived sorbitol to glycols over carbon-materials supported Ru-based catalysts. *Sci. Rep*. **5**, 16451; doi: 10.1038/srep16451 (2015).

## Supplementary Material

Supplementary Information

## Figures and Tables

**Table 1:** Hydrogenolysis of sorbitol over different ruthenium samples.

Entry	Catalyst	Conversion (%)	Yield based on carbon (%)				
			1,2-PD	EG	1,3-PD	1,2-BD	GLY
1	Ru/AC	88.6	24.8	16.7	0.3	0.9	4.3
2	Ru/CNTs	99.2	31.4	23.8	0.7	0.4	7.5
3	Ru/CNTs-in	98.3	28.5	18.4	0.8	0.6	8.7
4	Ru/CNTs-out	99.5	33.4	25.1	0.9	1.4	4.3
5	RuWO_x_/CNTs^a^	99.6	34.6	25.6	0.9	0.7	7.0
6	RuWO_x_/CNTs^b^	79.5	28.1	18.3	0.6	0.8	6.3

Reaction conditions: 10wt% D-sorbitol aqueous solution 25 g, catalyst 0.3 g, *n*(Ca(OH)_2_) = 1.7 mmol, 205 °C, 5.0 MPa H_2_, 2 h, 500 r/min. Note:

^a^*n*(WO_x_)/*n*(Ru) = 0.25.

^b^*n*(WO_x_)/*n*(Ru) = 0.50. 1,2-BD = 1,2-butanediol.

**Table 2:** Effect of base type on sorbitol hydrogenolysis.

Base	Conversion (%)	Yield (on a carbon basis, %)				
		1,2-PD	EG	1,3-PD	1,2-BD	GLY
None	44.9	2.7	2.5	0.8	0.7	4.0
Mg(OH)_2_	84.4	11.5	9.0	0.8	0.9	27.1
Ca(OH)_2_	99.2	31.4	23.8	0.7	0.4	7.5
Sr(OH)_2_	75.4	26.1	15.8	0.6	0.7	7.3
Ba(OH)_2_	66.1	30.9	18.8	0.9	0.7	9.5

Reaction conditions: 10wt% D-sorbitol aqueous solution 25 g, Ru/CNTs 0.3 g, *n*(OH^−^) = 3.4 mmol, 205 °C, 5.0 MPa H_2_, 2 h, 500 r/min.
