# Supplementary material for: Conversion of biomass-derived sorbitol to glycols over carbon-materials supported Ru-based catalysts
Source: Sci Rep. 2015 Nov 18;5:16451. doi: 10.1038/srep16451 (PMC4649675; doi:10.1038/srep16451)
Supplement: Supplementary Information [file srep16451-s1.doc]

**Supplementary information**

**Conversion of biomass-derived sorbitol to glycols over** **carbon-materials supported Ru-based catalysts**

Xingcui Guo, Jing Guan, Bin Li, Xicheng Wang, Xindong Mu * & Huizhou Liu *

*CAS Key Laboratory of Bio-based Materials, Qingdao Institute of Bioenergy and Bioprocess Technology, Chinese Academy of Sciences, Qingdao 266101, China.*

**Corresponding author. Fax: +86 53280662724; Tel: +86 53280662723*

*E-mail addresses: muxd@qibebt.ac.cn (X. Mu), liuhz@qibebt.ac.cn (H. Liu)*

**Table S1 Textural properties of the synthesized catalysts**

| Catalyst | S_BET_ ^a^  (m^2^ g^-1^) | N content ^b^  (wt%) | Ru content ^c^ (wt%) | W content ^c^  (wt%) | d_Ru_ ^d^  (nm) | Ru dispersion ^e^ (%) |
| --- | --- | --- | --- | --- | --- | --- |
| Ru/AC | 1160.1 | 0.75 | 3.99 | - | 2.70 | 18 |
| Ru/CNTs | 173.2 | - | 4.02 | - | 2.66 | 23 |
| Ru/CNTs-in | 138.2 | - | 4.05 | - | 1.97 | 28 |
| Ru/CNTs-out | 159.8 | - | 4.03 | - | 2.25 | 26 |
| RuWO_x_/CNTs ^f^ | 127.2 | - | 3.97 | 1.8 | 1.82 | 19 |
| RuWO_x_/CNTs ^g^ | 122.1 | - | 3.95 | 3.7 | 2.12 | 24 |

^a^ BET method; ^b^ Obtained from ICP analysis; ^c^ Obtained from ICP analysis; ^d^ Mean Ru particle size determined by TEM; ^e^ Ru dispersion obtained from H_2_ chemisorption; ^f^ *n*(WO_x_)/*n*(Ru) = 0.25; ^g^ *n*(WO_x_)/*n*(Ru) = 0.50.

**Table S2** Elemental analysis for activated carbons (AC) and carbon nanotubes (CNTs)

| Samples | C  (wt%) | N  (wt%) | S  (wt%) | H  (wt%) | O  (wt%) | Cl (wt%) | P (wt%) |
| --- | --- | --- | --- | --- | --- | --- | --- |
| AC | 92.75 | 2.52 | 0.08 | 3.01 | 1.47 | 0.03 | 0.04 |
| CNTs | 97.27 | 0.03 | 0 | 0.85 | 1.55 | 0 | 0 |

Note: The contents of C, N, S, H elements were determined by elemental analysis (CHNS/O Analyser); the Cl and P contents were determined by ICP-AES.


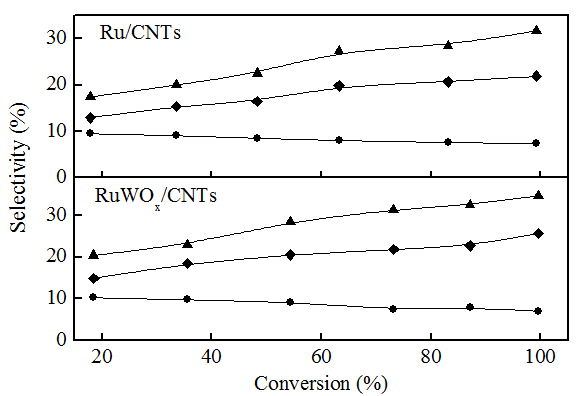


**Figure S1**. Dependence of product selectivities on sorbitol conversions over Ru/CNTs and RuWO_x_/CNTs. Selectivity to 1,2-PD (▲); Selectivity to EG (♦); Selectivity to GLY (●).

Reaction conditions: 205^o^C, 5.0 MPa H_2_, catalyst 0.3 g, 10 wt% D-sorbitol aqueous

solution 25 g, 0.1-2 h.


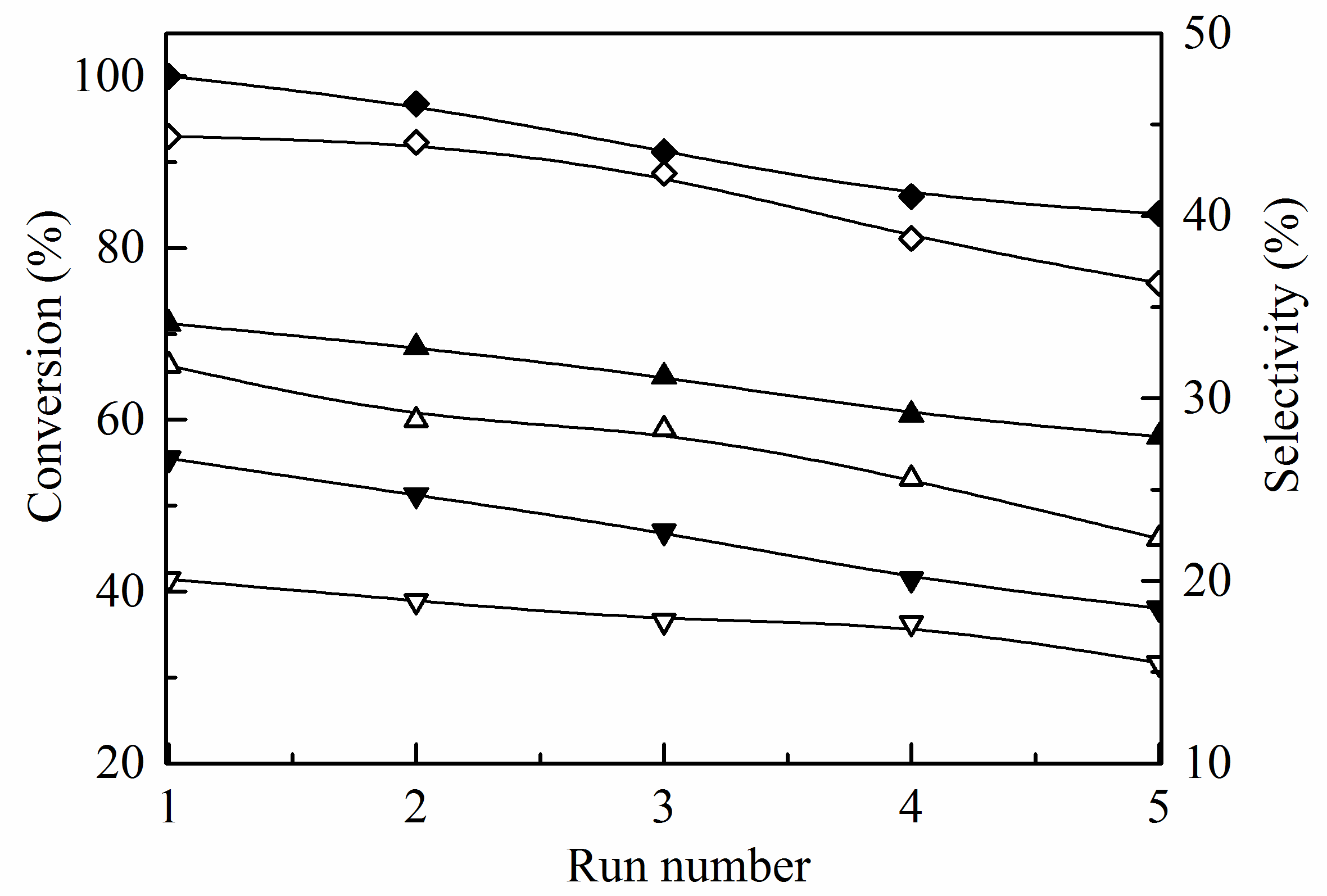


**Figure S2.** Stability of Ru/CNTs(◆, ▲, ▼) and RuWO_x_/CNTs (◇, Δ, ∇) catalysts upon recycling. Sorbitol conversion (◆, ◇); Selectivity to 1,2-PD (▲, Δ); Selectivity to EG (▼, ∇).

Reaction conditions: 205^o^C, 5.0 MPa H_2_, catalyst 0.3 g, 10 wt% sorbitol aqueous solution 25 g, 2 h.


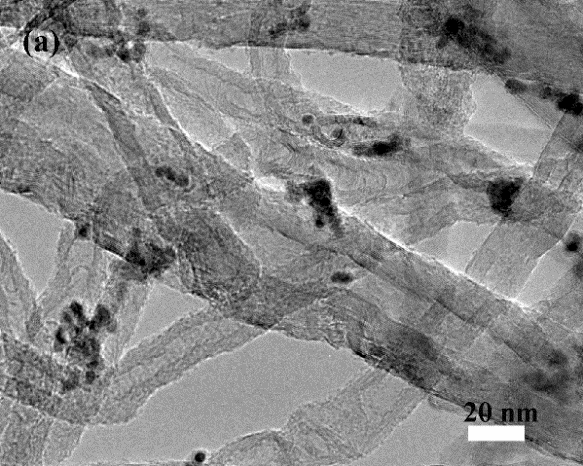
**
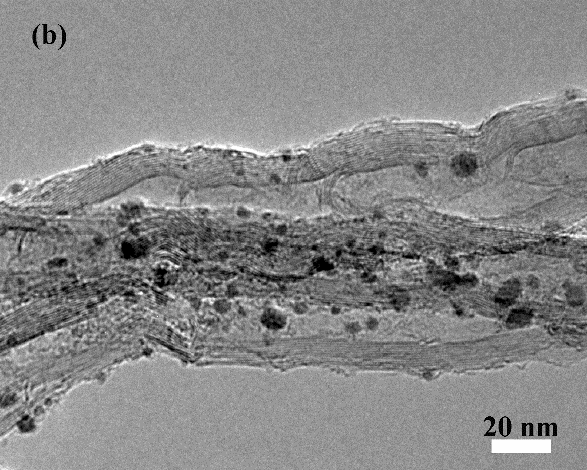
**


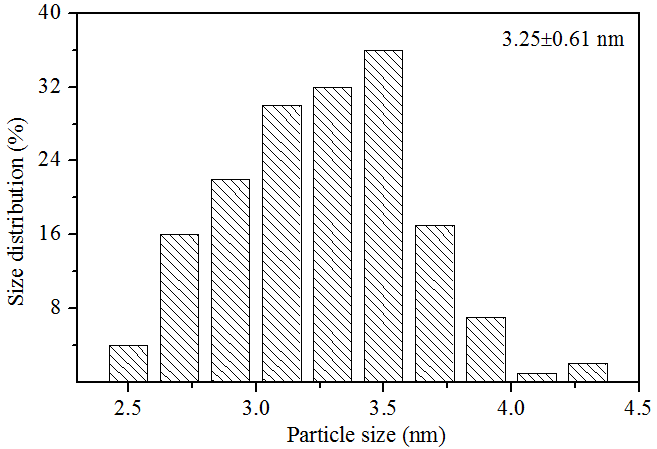

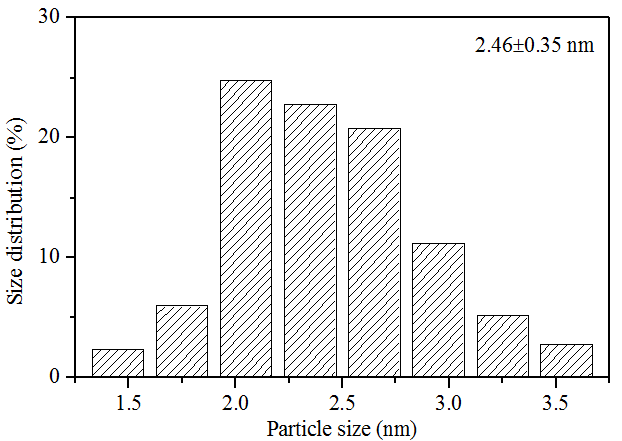


**Figure S3.** HRTEM images and ruthenium particle size distribution of catalysts (a) Ru/CNTs and (b) RuWO_x_/CNTs after the fifth run.
